# Supplementary material for: A Global Screen for Assembly State Changes of the Mitotic Proteome by SEC-SWATH-MS
Source: Cell Syst. 2020 Feb 26;10(2):133–155.e6. doi: 10.1016/j.cels.2020.01.001 (PMC7042714; doi:10.1016/j.cels.2020.01.001)

P23610 | F8I2_HUMAN | F8A1 F8A; F8A2 F8A; F8A3 F8A
Monomer MW [kDa]: 39.103 Monomer expected elution fraction: 48

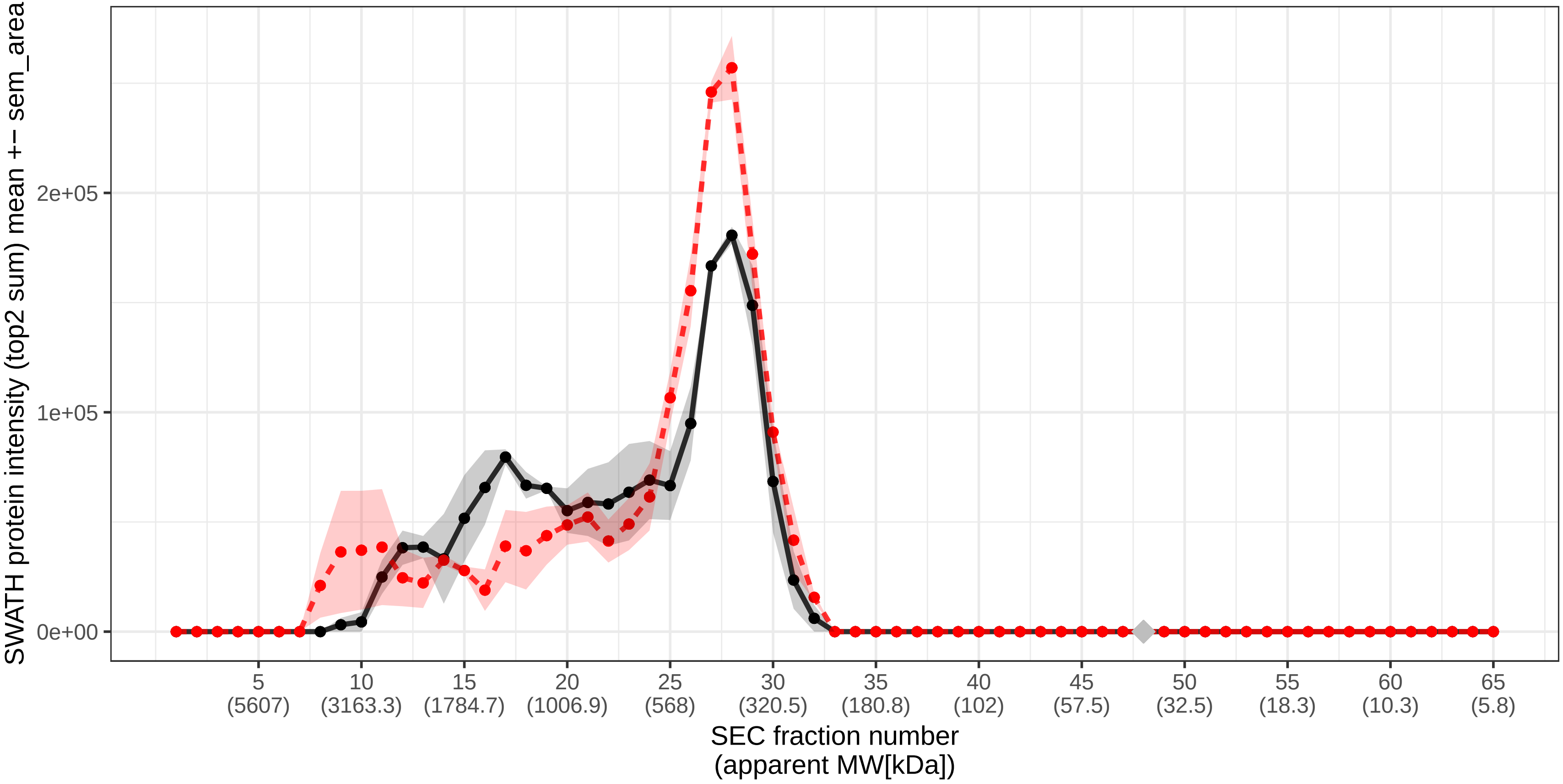

Supplement: Data S1. SEC-SWATH-MS Protein Chromatograms, Related to Figure 1 [file mmc6.zip › SECchrom_P23610_F8I2_HUMAN_F8A1_F8A;_F8A2_F8A;_F8A3_F8A.pdf]
